# Comparative effects of methamphetamine, cannabis, and polysubstance use on oral health

**DOI:** 10.3389/fpsyt.2025.1510228

**Published:** 2025-01-31

**Authors:** Osman Hasan Tahsin Kılıç, Duygu Kürklü Arpaçay, Onur Çağdaş Gezen, Zehra Nur Bayram, Aysel Başer

**Affiliations:** ^1^ Department of Psychiatry, Faculty of Medicine, Izmir Democracy University, Izmir, Türkiye; ^2^ Department of Prosthodontics, Faculty of Dentistry, Izmir Democracy University, Izmir, Türkiye; ^3^ Faculty of Dentistry, Izmir Democracy University, Izmir, Türkiye; ^4^ Department of Psychiatry, Health Science Institute, Izmir Democracy University, Izmir, Türkiye; ^5^ Department of Medical Education, Faculty of Medicine, Izmir Democracy University, Izmir, Türkiye

**Keywords:** substance use disorder, oral health behaviors, DMTF index, cannabis, methamphetamine

## Abstract

**Introduction:**

This study aimed to compare the clinical oral health status of individuals with Methamphetamine (MA), Cannabis (THC), and Polysubstance (PS) use disorders to healthy controls, and assess the impact of substance type, usage duration, quantity, and oral health behaviors on dental health outcomes through comprehensive oral examinations.

**Methods:**

This cross-sectional clinical study was conducted at İzmir Democracy University Buca Seyfi Demirsoy Education and Research Hospital between April 2024 and August 2024. A total of 190 individuals with substance use disorders (MA, THC, and PS users) and 91 healthy controls participated. Sociodemographic data, substance use history, and oral health behaviors were collected using a researcher-developed questionnaire and the Turkish version of the Hiroshima University Dental Behavior Inventory (HU-DBI). Oral health status was assessed through clinical oral examinations using the Decayed, Missing, and Filled Teeth (DMFT) index. Statistical analyses were performed using SPSS 26.0, applying t-tests, ANOVA, Chi-square tests, and Pearson correlation to evaluate group differences and relationships between variables. A p-value of < 0.05 was considered statistically significant.

**Results:**

The study revealed significant differences in oral health among substance users compared to the control group. MA users had the highest DMFT scores (11.04 ± 5.56), followed by THC users (9.49 ± 5.87), and PS users (8.40 ± 4.52), with the control group showing the lowest scores (6.08 ± 4.18) (p<0.001). The study also found a moderate positive correlation between MA use and DMFT scores, indicating that longer and higher usage leads to poorer oral health, while no significant association was observed between THC use and DMFT scores. Additionally, significant disparities in education levels were observed, with substance users having lower education compared to controls (p=0.001). HU-DBI scores indicated poorer oral health behaviors in substance users, though the difference was not statistically significant (p=0.053).

**Discussion:**

The study reveals that all substance use groups, including MA, THC, and PS users, exhibit significantly poorer oral health outcomes, with higher DMFT scores and worse oral health behaviors compared to the control group, highlighting the critical need for comprehensive dental care interventions for individuals with substance use disorders.

**Clinical Trial Registration:**

https://clinicaltrials.gov/study/NCT06640712, identifier NCT06640712.

## 1 Introduction

Substance Use Disorder (SUD) is a chronic and recurrent mental health condition that significantly affects the physical, psychological, and social dimensions of an individual’s life, as well as society (1, 2). Depending on the substance used, serious health complications such as stroke, cardiac arrhythmias, acute coronary syndrome, myocarditis, acute pulmonary edema, aortic dissection, seizures, respiratory depression, accidents, kidney failure, and even death may occur. Additionally, long-term substance use can impact all bodily systems and organs, including dental health, leading to disability, decreased quality of life, and increased mortality and morbidity (3).

Addictive substances can negatively impact oral health by directly damaging the physical structure of teeth, altering the immune system, or impairing the function of salivary glands (4). Additionally, low levels of oral health literacy, poor oral hygiene, and unhealthy diets among individuals with SUDs may exacerbate oral health problems (5). Furthermore, the effects of substances on brain function may lead to various maladaptive behaviors, such as risk-taking, aggression, and avoidance of dental health services, all of which contribute to worsening oral health outcomes in individuals with SUDs (6). Various substances may have specific effects on teeth. However, comparative research on the oral health effects of substances is limited. Moreover, most research on SUDs has focused on single substances in isolation, often excluding individuals with a history of polysubstance use from clinical studies (7, 8). Yet, many individuals with SUDs engage in polysubstance use. For example, a study from our country revealed that the prevalence of single substance use is 4.5%, while multiple substance use is 2.6% (2).

This study aims to compare the clinical oral health status of individuals with MA, THC, and PS use disorders to healthy controls, and assess the impact of substance type, usage duration, quantity, and oral health behaviors on dental health outcomes through comprehensive oral examinations.

## 2 Materials and methods

### 2.1 Ethical considerations

The research protocol was approved by the Ethics Committee of İzmir Democracy University Buca Seyfi Demirsoy Education and Research Hospital (approval number 2024/234). All participants were informed about the purpose of the study and provided written informed consent. The study was conducted in full compliance with the Declaration of Helsinki. This study utilized a cross-sectional, case-control design.

### 2.2 Study design

**Figure 1:**
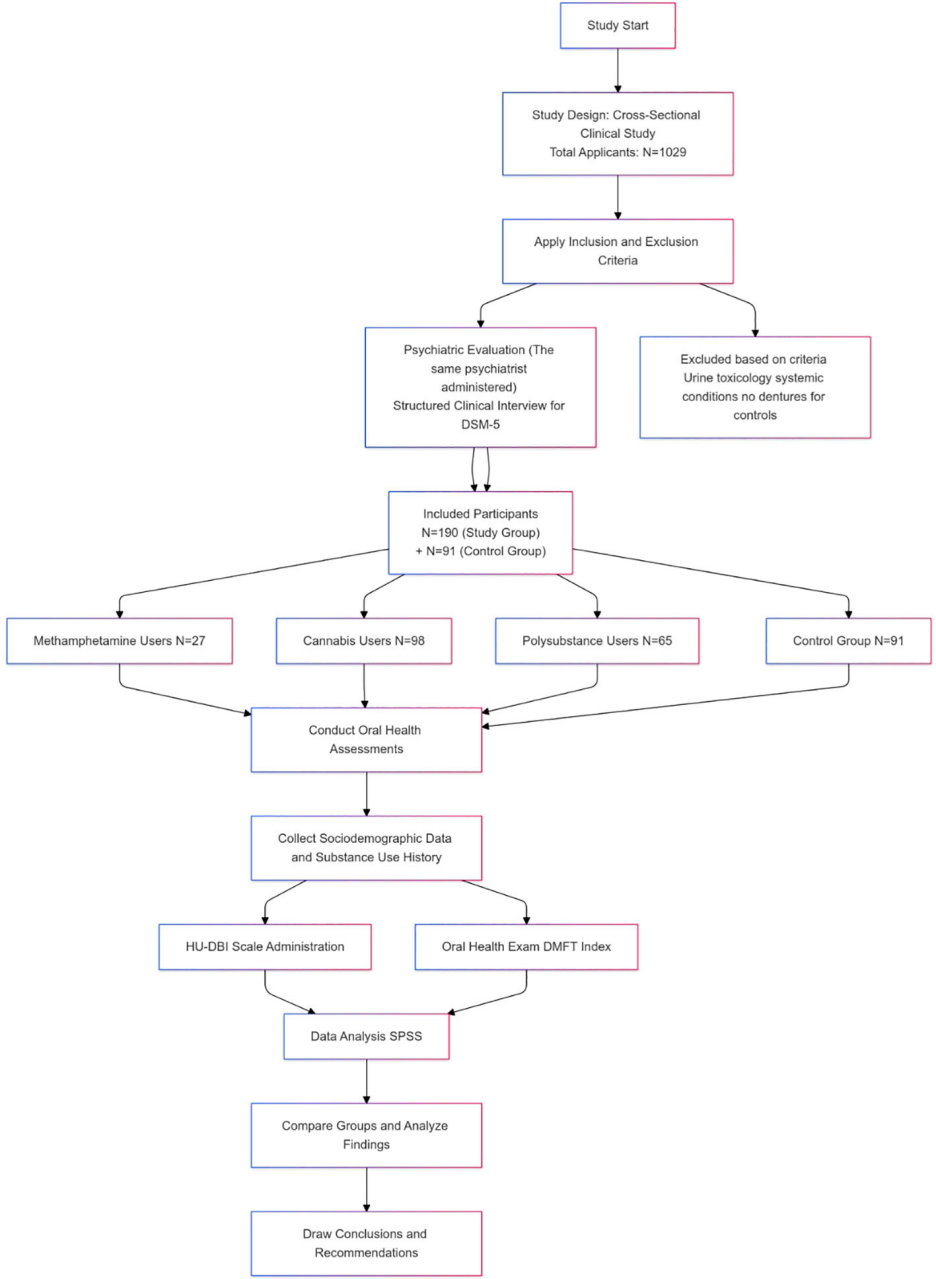
Study flow chart.

The design of the study was a cross-sectional clinical study. This cross-sectional clinical study aimed to compare the oral health status of individuals with MA, THC, and Polysubstance use disorders to healthy controls. The study included clinical oral examinations to assess participants’ oral health using the Decayed, Missing, and Filled Teeth (DMFT) index, along with self-reported oral health behaviors. The clinical oral examinations were conducted at İzmir Democracy University Buca Seyfi Demirsoy Education and Research Hospital Probation Clinic between April 2024 and August 2024. In Turkey, individuals involved in the use, purchase, or possession of controlled substances are mandated to attend probation clinics, following national policy. A total of 1,029 individuals applied to the probation clinic during the study period (**Figure 1**).

A statistical power analysis using G*Power was performed to determine the required sample size for the study. Assuming 80% statistical power (1-β error probability) and a 5% margin of error (α error probability), the calculation indicated a required sample size of approximately 51 participants per group. The study ultimately included a sample size of 190 participants in the study group (substance users) and 91 in the control group, which was sufficient to detect medium effect sizes (Cohen’s d = 0.5) with 80% power. All participants provided informed consent.

### 2.3 Subjects and clinical assessments

Dental and psychiatric evaluations were conducted at the Probation Clinic of İzmir Democracy University Buca Seyfi Demirsoy Education and Research Hospital. The same psychiatrist administered the Structured Clinical Interview for Diagnostic and Statistical Manual of Mental Disorders, Fifth Edition (DSM-5) to all participants (9). Additionally, sociodemographic data and responses to the Hiroshima University Dental Behavioral Inventory (HU-DBI) scale were collected.

Oral health examinations were performed by a trained dental student and a specialist dentist using a portable dental chair, artificial light, and a dental mirror. Dental caries was assessed using the DMFT index (Decayed, Missing, and Filled Teeth) according to the World Health Organization’s caries diagnostic criteria (10). This index included the number of decayed, missing (due to caries, excluding 3rd molars), and filled teeth in the permanent dentition. The oral health examiners were blinded to each participant’s substance use type and sociodemographic information.

The study group was divided into three subgroups based on the type of substance used. Participants who used only MA, only THC, or both substances together (PS group) were included in the study group. The control group consisted of male hospital employees, including secretaries, cleaning staff, ad security personnel, who had no history of substance use, systemic medical conditions, or medication use (**Figure 1**).

### 2.4 Inclusion/exclusion criteria

Male participants aged 18 to 45 who used MA, THC, or both were included in the study group. The exclusion criteria were absence of urine toxicology analysis results, use of substances other than MA, THC, or both, being under the influence of substances during the evaluation, not meeting DSM-5 criteria for Substance Use Disorder, presence of systemic medical conditions, current medication use, or incomplete scales and forms. In the control group, individuals with a history of substance use, systemic medical conditions, or medication use were excluded. Additionally, participants who wore removable dentures were also excluded.

### 2.5 Assessment tools

#### Sociodemographic data form

This form consists of 8 questions developed by the researchers, covering information such as age, education level, marital status, living situation, employment status, health insurance status, type and quantity of substance use, duration of use.

#### Hiroshima University Dental Behavior Inventory

Participants’ oral health attitudes and behaviors were assessed using the Turkish version of the HU-DBI questionnaire (11). The HU-DBI, developed by Kawamura (12, 13), consists of 20 items in a dichotomous response format (agree/disagree). One point is given for each “agree” response to items 4, 9, 11, 12, 16, and 19, and one point is given for each “disagree” response to items 2, 6, 8, 10, 14, and 15. The maximum possible score is 12, and the minimum score is 0. Higher scores indicate better oral health attitudes and behaviors.

## 3 Statistical analysis

Statistical analyses were conducted using SPSS (Statistical Package for the Social Sciences) version 26.0. Descriptive statistics, t-tests, ANOVA, and Pearson correlation analyses were employed. Frequencies and percentages were calculated for categorical variables (marriage status, employment status, living with having health insurance, used substance, disease severity). Means (M) and standard deviations (SD) were reported for continuous variables (age, education level, substance use index, DMFT scores, and HU-DBI scale scores). Independent-samples t-tests were used to compare substance use and control group DMFT and HU-DBI scores. One-way ANOVA was employed to examine differences in age, education, DMFT and HU-DBI scores across different substance use groups (MA, THC, PS). Significant differences identified by ANOVA were further explored using Bonferroni *post-hoc* tests. The distribution of categorical variables across the groups was evaluated using the Chi-square test to determine if there were significant differences between the groups. Pearson correlation analysis was performed to assess relationships among continuous variables, including substance use index, DMFT, and HU-DBI scores. Correlation coefficients (r) and p-values were reported. p < 0.05 was considered statistically significant.

## 4 Results

The results section presents an analysis of the study’s findings, including sociodemographic variables, substance use-related data, oral health and behaviors, and correlations between substance use and oral health outcomes.

### 4.1 Sociodemographic variables

The study included four groups: MA users (n=27), THC users (n=98), PS users (n=65), and a control group (n=91). The mean age was 32.67 ± 7.86 years in the MA group, 30.97 ± 10.71 years in the THC group, 29.05 ± 6.22 years in the PS group, and 30.91 ± 6.95 years in the control group. There was no statistically significant difference in age between the four groups (p=0.248). The control group had the highest mean years of education at 12.69 ± 3.67 years, followed by the THC group with 9.44 ± 3.36 years, the PS group with 9.38 ± 2.63 years, and the MA group with 7.93 ± 2.93 years. The difference in education years was statistically significant between the four groups but not among the substance use groups themselves (p=0.001). Most participants in all groups were single: 16 participants (59.2%) in the MA group, 71 participants (72.4%) in the THC group, 46 participants (70.7%) in the PS group, and 58 participants (63.7%) in the control group. There was no statistically significant difference in marriage status between the four groups (p=0.419). In the MA group, 26 participants (96.2%) resided alone, with only 1 participant (3.7%) living with family or friends. Similarly, in the THC group, 81 participants (82.6%) lived alone, while in the PS group, 56 participants (86.1%) lived alone. In comparison, 64 participants (70.3%) in the control group lived alone. However, no statistically significant difference in living arrangements was found among the four groups (p=0.102).

In the MA group, 14 participants (51.8%) had health insurance, compared to 66 participants (67.3%) in the THC group and 42 participants (64.6%) in the PS group. The differences in health insurance coverage among the SUD groups were not statistically significant (p = 0.330). The control group, comprised entirely of hospital staff who all had health insurance, was excluded from this analysis. Regarding employment status, 19 participants (70.3%) in the MA group were employed, along with 70 participants (71.4%) in the THC group, and 52 participants (80%) in the PS group. These differences in employment status were also not statistically significant (p = 0.418). The control group was also not included in this analysis because its members were hospital staff. The sociodemographic characteristics of the participants are presented in **Table 1**.

**Table 1:** Socio-demographic characteristics of the participants.

	MA (n=27)	THC (n=98)	PS (n=65)	CONTROL (n=91)	p
**Age (mean ± sd)**	32.67 ± 7.86	30.97 ± 10.71	29.05 ± 6.22	30.91 ± 6.95	0.248
**Education years (mean ± sd)**	7.93 ± 2.93	9.44 ± 3.36	9.38 ± 2.63	12.69 ± 3.67	**0.001^1^**
**Marriage status n (%)** Married Single	11(40.7%) 16(59.2%)	27(27.5%) 71(72.4%)	19(29.2%) 46(70.7%)	33(36.2%) 58(63.7%)	0.419
**Living with n (%)** Alone Family/friends	26(96.2%) 1(3.7%)	81(82.6%) 17(17.3%)	56(86.1%) 9(13.8%)	64(70.3%) 21(29.6%)	0.102
**Health Insurance n (%)** Yes No	14(51.8%) 13(48.1%)	66(67.3%) 32(32.6%)	42(64.6%) 23(35.3%)		0.330
**Employment n (%)** Yes No	19(70.3%) 8(29.6%)	70(71.4%) 28(28.5%)	52(80%) 13(20%)		0.418

**^1^** The difference was between control and SUD (MA, THC, PS) groups. There was no difference between SUD groups.

^2^The bold values indicate statistically significant differences.

### 4.2 Substance use disorder severity across groups

In the MA group, disease severity was classified as mild in 7 individuals (25.9%), moderate in 4 individuals (14.8%), and severe in 16 individuals (59.2%). In the THC group, disease severity was classified as mild in 29 individuals (29.5%), moderate in 12 individuals (12.2%), and severe in 57 individuals (58.1%). In the PS group, disease severity was classified as mild in 8 individuals (12.3%), moderate in 8 individuals (12.3%), and severe in 49 individuals (75.3%). No significant difference was found in disease severity between SUD groups (p=0.126).

### 4.3 Oral health examination and oral health behaviors

The mean DMFT score was 11.04 ± 5.564 in the MA group, 9.49 ± 5.875 in the THC group, 8.40 ± 4.520 in the PS group, and 6.08 ± 4.180 in the control group. There was a significant difference in DMFT scores between the SUD groups and the controls (p=0.001); however, no significant difference was found among the substance use groups (p>0.05). The proportions and counts of DMFT scores categorized according to WHO classifications (very low, low, moderate, high) were as follows: in the MA group, 4 (14.8%) participants had very low scores, 6 (22.2%) participants had low scores, 10 (37.1%) participants had moderate scores, and 7 (25.9%) participants had high scores; in the THC group, 21 (21.4%) participants had very low scores, 27 (27.5%) participants had low scores, 31 (31.6%) participants had moderate scores, and 19 (19.4%) participants had high scores; in the PS group, 11 (16.9%) participants had very low scores, 26 (40.0%) participants had low scores, 21 (32.3%) participants had moderate scores, and 7 (10.7%) participants had high scores; and in the control group, 35 (38.5%) participants had very low scores, 37 (40.7%) participants had low scores, 12 (13.2%) participants had moderate scores, and 7 (7.7%) participants had high scores. These differences in DMFT distribution among the groups were statistically significant (p = 0.001), and detailed explanations are provided in **Table 2**.

**Table 2:** Clinical characteristics of the participants.

	MA (n=27)	THC (n=98)	PS (n=65)	CONTROL (n=91)	p
**DMFT (mean ± sd)**	11.04 ± 5.56	9.49 ± 5.87	8.40 ± 4.52	6.08 ± 4.18	**0.001^1^**
**DMFT (according to WHO)** **very low** **low** **moderate** **high**	4 (14.8%) ^b^ 6 (22.2%) ^a^ 10 (37.1%) ^b^ 7 (25.9%) ^b^	21 (21.4%) ^b^ 27 (27.5%) ^a^ 31(31.6%) ^b^ 19 (19.4%) ^b^	11 (16.9%) ^b^ 26 (40.0%) ^a^ 21(32.3%) ^b^ 7 (10.7%) ^a,b^	35 (16.9%) ^a^ 37 (16.9%) ^a^ 12 (16.9%) ^a^ 7 (16.9%) ^a^	**0.001^2^**
**HU-DBI (mean ± sd)**	4.52 ± 1.94	5.40 ± 1.71	6.65 ± 1.807	5.30 ± 1.79	0.053
**Disease Severity n (%)** **Mild** **Moderate** **Severe**	7 (25.9%) 4 (14.8%) 16 (59.2%)	29 (29.5%) 12 (12.2%) 57 (58.1%)	8 (12.3%) 8 (12.3%) 49 (75.3%)		0.126
**daily dosage (gram) x year**	4.139 ± 8.86	8.519 ± 19.76			

**^1^** The difference was between control and SUD (MA, THC, PS) groups. There was no difference between SUD groups.

**^2^** Groups sharing the same letter (a, b, c) do not have a statistically significant difference. Groups with different letters are significantly different from each other.

^3^The bold values indicate statistically significant differences.

The mean HU-DBI score was 4.52 ± 1.949 in the MA group, 5.40 ± 1.716 in the THC group, 6.65 ± 1.807 in the PS group, and 5.30 ± 1.792 in the control group. The difference in HU-DBI scores between the four groups did not reach statistical significance (p=0.053). The psychiatric and dental clinical data of the participants are presented in **Table 2**.

### 4.4 Correlation analysis

The MA index (daily dosage (gram) x duration of use (year)) was 4.139 ± 8.86 and The THC index was (daily dosage (gram) x duration of use (year)) 8.519 ± 19.76. The MA index was moderately and positively correlated with the DMFT score (p=0.036, r=0.405). However, no significant correlation was found between the THC index and the DMFT score (p=0.930, r=0.009).

## 5 Discussion

This study is the first to examine the comparative effects of MA, THC, and polysubstance use on oral health through both clinical oral and psychiatric evaluations. Our findings indicate that oral health status is significantly poorer in all three Substance Use Disorder groups compared to healthy controls. However, there were no significant differences in oral health outcomes between those using MA, THC, or PS. Our findings suggest that each of the substances examined contributes to the deterioration of oral health. These results align with the findings by D’Amore, who also observed that substance use, did not show association with self-reported oral health status when comparing different substances (14). Notably, both studies emphasize the significant overall decline in oral health among individuals with SUD, regardless of the specific substance used.

In recent years, studies investigating the effects of substances on oral health have primarily focused on MA users (15, 16). Several case reports have described a condition referred to as “Meth mouth,” characterized by teeth that are “blackened, stained, rotting, crumbling, or falling apart” (17, 18). However, our findings suggest that the overall dental health of MA users may not be as severely affected as these case reports suggest. Epidemiological studies also indicate that such extreme presentations are not typical, with average Decayed Teeth (DT) scores ranging from 2 to 5, and Missing Teeth (MT) scores between 3 and 5 (19–21). This aligns with our results, showing that while MA use has a noticeable impact on oral health, the severe cases of “Meth mouth” may be exceptions rather than the rule. In the literature, MA use, in particular, is associated with high DMFT (Decayed, Missing, and Filled Teeth) scores, linked to severe tooth decay, xerostomia (dry mouth), and poor oral hygiene. Lower education levels, higher rates of living alone, and lack of consistent access to healthcare services also contribute to poor oral health outcomes in individuals who use these substances (19). In this study, it was found that individuals with substance use disorders had worse oral health compared to healthy controls. MA users exhibited the highest DMFT scores, followed by THC and PS users. Additionally, socio-demographic factors such as lower education levels and higher rates of living alone contributed to poor oral health. In line with the literature, our findings emphasize the critical need for targeted interventions focusing on oral health for individuals with substance use disorders.

Regarding THC use, previous studies have identified the primary dental complication as an increased incidence of dental caries. This is considered to be linked to poor oral hygiene, higher plaque scores, and reduced saliva production associated with THC use (22). In line with these studies, which reported higher DMFT (Decayed, Missing, and Filled Teeth) scores among THC users (23, 24), we also found that THC users had significantly higher DMFT scores compared to controls.

Studies exploring the effects of polysubstance use on dental health are limited. One study examining substances used alongside cocaine found that periodontitis was the most prevalent condition among individuals who used two or more substances in addition to cocaine. Additionally, it has been reported that untreated dental decay is the primary dental health issue among those who use both cocaine and MA (25). In another study focusing on the oral health of polysubstance users, dental caries and tooth loss were identified as the most common issues. However, that study included individuals using various substance combinations (e.g., tobacco, alcohol, THC, cocaine) and did not compare them to healthy individuals. It also found that those using drugs for over a year had a higher likelihood of developing dental caries (26). Similarly, in our study, we used MA and THC indices (daily dosage (gram) x year) to assess the impact of the duration and type of drug use on oral health. We found a positive and moderate correlation between the MA index and DMFT scores, suggesting that longer duration and higher amounts of MA use are associated with poorer oral health. However, we did not find any association between THC index and DMFT scores. This difference may be related to the direct dose- and duration-dependent destructive effects of MA, whereas THC affects oral health primarily through promoting plaque formation and reducing saliva production (22).

Previous studies have reported that oral health behaviors among substance users are inadequate (27, 28). However, in our study, no significant differences were found between the SUD groups and the control groups in terms of oral health attitudes and behaviors as evaluated by HU-DBI scores. This may be linked to the overall poor oral health behaviors observed in the general population of Türkiye. According to the Türkiye Oral and Dental Health Survey, approximately 6-10% of adults in Türkiye never brush their teeth (29). Additionally, annual toothpaste consumption in Türkiye is 110 grams, which is considerably lower compared to developed countries (29). Therefore, dentists should inquire about substance use in every patient and, if detected, encourage and refer them to substance use disorder treatment. Although MA has recently been in the spotlight due to its striking dental complications, THC remains the most widely used illicit substance worldwide (30). Moreover, it has been legalized in some countries, such as Germany and Canada. Additionally, the trend of multi-substance use continues to increase steadily (7). Therefore, it is crucial for dentists not to overlook these conditions.

Future studies should focus on the development of comprehensive, interprofessional educational programs that address both dental and substance use treatment needs. Given the complexity of oral health complications arising from polysubstance use, it is essential to foster collaboration between dentists and mental health professionals. Interprofessional education programs can be designed to train healthcare providers in the early identification and management of substance use disorders, integrating dental care into substance use disorder treatment pathways. Such programs should emphasize the role of dentists in recognizing signs of substance abuse and highlight the importance of referral systems to ensure patients receive holistic care. Additionally, research should explore the impact of these interdisciplinary approaches on patient outcomes, particularly in settings where THC and other substances are becoming more legalized.

This study has some limitations. First, women were not included in the study due to their low representation in probation clinics in Turkey, where only three percent of referrals are women, making it challenging to obtain a representative sample (31). Second, although substance use was objectively confirmed through urine tests, the duration and quantity of substance use relied on self-reported data. Such data can be prone to recall bias or underreporting, particularly in populations with substance use disorders.

In summary, this study highlights the importance of addressing oral health as part of comprehensive care for individuals with substance use disorders. The significant differences in oral health outcomes between substance users and healthy controls underscore the need for early interventions and continuous care to reduce the burden of dental diseases in this vulnerable population. Future studies could benefit from incorporating additional oral health indicators, such as salivary flow rate, oral hygiene index, and periodontal health metrics (gingival index and alveolar bone loss) alongside the DMFT index to provide a more comprehensive evaluation of oral health status.

In conclusion, while MA use clearly has the most severe impact on oral health, this study provides valuable insights into the broader spectrum of oral health issues faced by individuals who use multiple substances such as THC and PS, emphasizing the need for comprehensive dental interventions in substance use disorder treatment. Our findings have shown that individuals with substance use disorders have poorer oral health. Specialists in substance use disorder treatment should be aware of the oral health problems present in these patients. Addressing oral health issues may enhance patients’ adherence to substance use disorder treatment and increase their motivation, contributing to better overall outcomes.

### 5.1 Practical implications

As highlighted in the literature, It is not only MA that leads to severe dental problems, but other forms of substance use have detrimental effects on oral health. Substance use, including MA, THC, and PS, significantly compromises oral hygiene and health. Therefore, addressing the oral health needs of this population requires a comprehensive, multidisciplinary approach. Integrating dental care into broader substance use disorder treatment programs is essential to meet both the acute and chronic oral health needs of these individuals. Prevention and education on oral hygiene, combined with accessible dental care, are critical to mitigating the serious oral health impacts of substance use. By fostering interprofessional collaboration among dentists and mental healthcare providers, targeted interventions can be developed to reduce the burden of dental diseases in substance users. This collaborative approach is vital for ensuring long-term improvements in both oral health and overall recovery outcomes for individuals with substance use disorders.

## Data Availability

The original contributions presented in the study are included in the article/**Supplementary Material**. Further inquiries can be directed to the corresponding author.
